# Effect of cartilaginous rings in tracheal flow with stenosis

**DOI:** 10.1186/s42490-023-00068-4

**Published:** 2023-06-01

**Authors:** Humberto Bocanegra Evans, Jose Montoya Segnini, Ali Doosttalab, Joehassin Cordero, Luciano Castillo

**Affiliations:** 1 School of Mechanical Engineering, Purdue University, 1500 Kepner Dr Room 104, West Lafayette, IN 47905 USA; 2 Department of Otolaryngology, Texas Tech University Health Sciences Center, Lubbock, TX 79430 USA

**Keywords:** Particle image velocimetry, Tracheal stenosis, Refractive index-matching, Flow separation, Respiration

## Abstract

**Background:**

In respiratory fluid dynamics research, it is typically assumed that the wall of the trachea is smooth. However, the trachea is structurally supported by a series of cartilaginous rings that create undulations on the wall surface, which introduce perturbations into the flow. Even though many studies use realistic Computer Tomography (CT) scan data to capture the complex geometry of the respiratory system, its limited spatial resolution does not resolve small features, including those introduced by the cartilaginous rings.

**Results:**

Here we present an experimental comparison of two simplified trachea models with Grade II stenosis (70% blockage), one with smooth walls and second with cartilaginous rings. The use a unique refractive index-matching method provides unprecedented optical access and allowed us to perform non-intrusive velocity field measurements close to the wall (e.g., Particle Image Velocimetry (PIV)). Measurements were performed in a flow regime comparable to a resting breathing state (Reynolds number Re_*D*_ = 3350). The cartilaginous rings induce velocity fluctuations in the downstream flow, enhancing the near-wall transport of momentum flux and thus reducing flow separation in the downstream flow. The maximum upstream velocity in the recirculation region is reduced by 38%, resulting in a much weaker recirculation zone— a direct consequence of the cartilaginous rings.

**Conclusions:**

These results highlight the importance of the cartilaginous rings in respiratory flow studies and the mechanism to reduce flow separation in trachea stenosis.

## Background

Even though the trachea and main bronchi have undulated walls as a result of the cartilaginous rings [26, 33], most research in respiratory fluid dynamics omits these features under the assumption that their effect is negligible. Nevertheless, existing research points to the importance of these small protrusions in both flow structure and aerosol deposition [6, 27, 33]. It is well established that surface roughness can have significant impact on wall-bounded flows [16], particularly when an adverse pressure gradient (APG), for example, an expansion in the cross-sectional area, is present [30]. This situation is regularly encountered in the respiratory tract, for instance, when tracheal stenosis is present. Sufficiently strong APGs can induce flow separation, which can have negative effects such as flow blockage [43]. Unfortunately, little information is available on the effects of cartilaginous rings under adverse pressure gradient conditions; more specifically, on the effect these structures have on the flow through a stenosed trachea. The objective of this study is to understand the effect of cartilaginous rings on the flow passing through a stenosed trachea.

Accurate knowledge of the flow characteristics in the respiratory system is vital for medical applications. For example, the development of diagnosis and treatment protocols for different diseases, pollutants and drug delivery. However, due to the complexity of the flow in the respiratory tract, simplified models have been developed to understand the characteristics of the flow. A consistent simplification in respiratory fluid dynamics research is the omission of the undulations introduced by the cartilage rings, that is, the use of smooth walls [1, 3, 4, 9, 10, 22, 24, 32, 45].

In recent years, scientists have generated increasingly realistic models using Computed Tomography (CT) scan [12, 13, 15, 18, 23, 29]. Although CT scans capture many of the complex geometries, they have a spatial resolution of 0.5–0.625 mm [21], whereas the thickness of the rings is approximately 0.25 mm [33].

The few studies that have evaluated the effects introduced by cartilaginous rings have found increased particle deposition [33, 44], increased shear over the ring surface [14, 39], reduced flow separation in the bifurcation [6], flow recirculation in the ring cavity [27], and flow asymmetry into the lungs [14].

The obstruction in the airway ducts considerably affects the flow dynamics and the mechanics of drug delivery [7, 11, 40, 41]. Tracheal stenosis is characterized by the narrowing of the tracheal lumen. It can be a congenital or acquired due to complications of endotracheal intubation and tracheostomy [5, 38]. Patients with airway narrowing usually present different symptoms including stridor, shortness of breath, wheezing, coughing, respiratory distress or pneumonia. Often, at the point of admission to the clinic, patients present loss of more than 75% of lumen (severe area contraction) [34]. Beside tracheal stenosis there are other diseases that cause a contraction in the airway ducts, e.g. trachea-bronchomalacia and excessive dynamic airway collapse [5, 28].

Few numerical studies have been carried out trying to elucidate the flow characteristics in such geometries; none has included cartilaginous rings [31, 42, 46, 47]. Brouns et al. [7] showed a pressure drop in the normal breathing which was only observed in cases where severe tracheal narrowing had occurred, i.e. greater than a 70% of obstruction. Hence, according to the simulations the detection of pre-critical stages of stenosis is hard to obtain by pressure differences due to only being affected at severe constriction. Similarly, Johari et al. [17] evaluated the effect of the location along the trachea of the stenosis, determining that higher pressure and flow dynamics differences were obtained as the stenosis gets closer to the bifurcation regions.

Another numerical study, by Taherian, Rahai, Gomez, et al. [41], demonstrated that treatment by stent for excessive dynamic airway collapse improved the breathing conditions, although this was not detected in a spirometry test. Additionally, a study by Taherian, Rahai, Bonifacio, et al. [40], which included experimentally-validated numerical simulations, showed a pressure drop and an increased particle deposition downstream of the stenosis.

In the present study we will assess the effect of cartilage rings on the flow through a stenosed trachea (grade II, 70% blockage). We analyze in detail the effect of the cartilaginous rings near the wall and its effect on flow separation through a cross-sectional area contraction, and expansion. It is the aim of this study to verify our hypothesis that the presence of cartilaginous rings (roughness) in a stenosed trachea will introduce flow perturbations that will reduce flow separation in the downstream side of the contraction.

## Methods

### Experimental setup

**Fig. 1:**
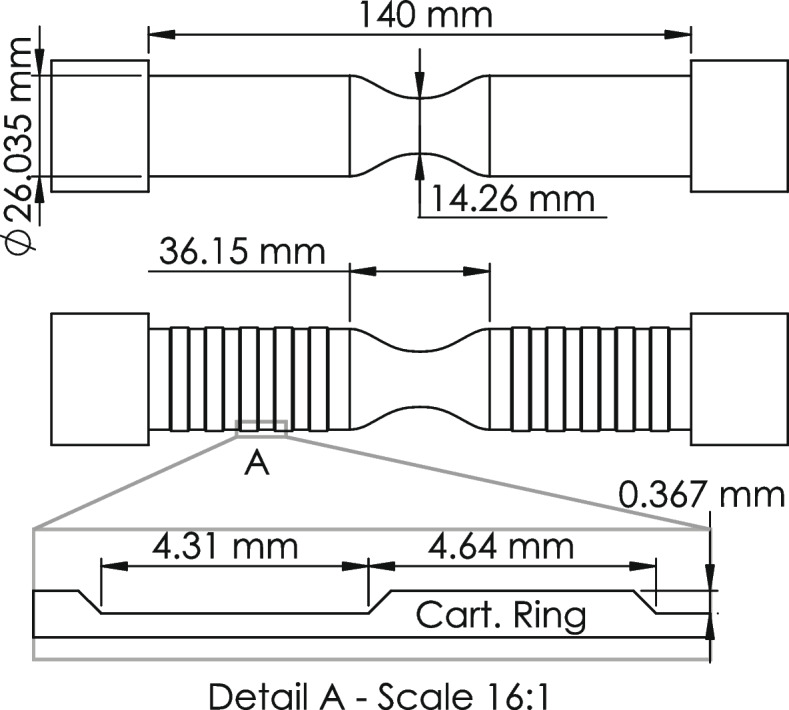
Trachea models dimensions for smooth (top) model and model with cartilaginous rings (bottom)

We measured the flow inside two simplified trachea models with two-dimensional particle image velocimetry (PIV) [2]. Both models have a circular cross-sectional area and an axisymmetric contraction of 70% at the middle of the model. Such contraction allows to observe the effect of tracheal stenosis as well as observe the effect on flow separation, which previous studies have demonstrated to be affected by the rings [6, 27]. The 70% area reduction was selected in the hope of understanding why the diagnosis of trachea stenosis below 75% area reduction remains a challenge [35]. One model has a smooth wall and the other model has idealized symmetric rings, simulating the presence of cartilaginous rings, as shown in Fig. 1.

The diameter of a typical human trachea is 18 mm [25] and the average ring thickness, width and separation between rings are 0.254 mm, 3.21 mm and 2.98 mm, respectively [33]. In our models, dimensions are scaled up 44% to increase the resolution of the measurements. Resulting in a diameter *D* = 26.0 mm for both models, a contraction diameter *d*_*c*_ = 14.3 mm, and for the ‘ringed’ model a ring thickness *t*_*R*_ = 0.37 mm, ring width *w*_*R*_ = 4.6 mm and distance between rings *w*_*C*_ = 4.3 mm. The models were created from a transparent silicone (polydimethylsiloxane or PDMS) and tested using a solution of water-glycerin-salt (47.9–37.1%-15%, respectively) as working fluid [36]. The models were submerged in a tank with the same fluid and the solution was made to match the refractive index of the PDMS (*n* = 1.42), thus providing optical access to the flow inside the model, along with preventing light reflections when capturing images near the wall. The experiments were performed at the room temperature of 21 °C. The flow rate was based on a resting breathing state with Reynolds number, Re_D_ = 3350, where the Reynolds number is a scaling non-dimensional parameter and is defined as the ratio of inertial to viscous forces,

$$\mathit{\operatorname{Re}}=\frac{DU}{\upsilon },$$

where the kinematic viscosity of the working fluid is *υ* = 5.77 × 10^− 6^ m^2^/s, the bulk velocity is *U* = 0.76 m/s (equivalent to a flow rate *Q* = 24 mL/min) and the previous mentioned diameter *D* = 26.035 mm. The density of the solution is *ρ* = 1080 kg/m^3^. Hence, by matching the Reynolds number we ensure that the flow behavior is dynamically the same as that observed in an air flow passing through the human trachea.

**Fig. 2:**
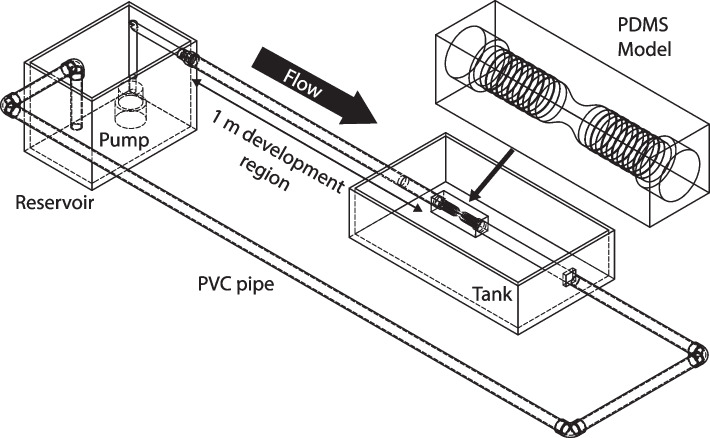
Experimental setup for showing the flow circuit, tank and PDMS model. We use Particle Image Velocimetry (PIV) to characterize the flow

A submerged pump with power rating of 1/6 hp is used to supply a continuous inspiratory flow to the trachea model, as observed in Fig. 2. The flow entering the trachea is expected to be fully developed, as a 1-m long development region was set before the model to allow for consistency during different trials and avoid flow irregularities from the pump. To allow for a detailed comparison between the two models, all conditions were kept the same.

The models were designed in the 3-D modeling software (SolidWorks). Afterward, we 3D printed the models in a water-soluble material called PVA (polyvinyl alcohol) with an Original Prusa I3 MK2 3D printer. The 3-D printed models are placed inside an acrylic container and the PDMS is poured in the container with the PVA mold. Once the PDMS is cured, the model is taken out of the acrylic container and is submerged in water for the water to dissolve the PVA mold.

### Measurement technique

We used a 2D planar PIV system to analyze the velocity field. The PIV system consist of an 8-bit CCD camera with resolution of 4008 × 2672 pixel^2^ and a Nd:YAG 532 nm laser to illuminate the tracing particles. The fluorescent particles used are made of a polyamide and have a diameter of 15 μm and density of *ρ* = 1100 kg/m^3^ (Kanomax, New York). As explained in a previous study, the particles can be considered tracers that accurately follow the flow [27]. We used lenses to create a thin laser sheet of 1 mm to illuminate the particles, the thin sheet is located at the center of the model along the streamwise direction. We captured a window before and after the contraction (including the contraction in both cases) with a resolution of 59.7 pixels/mm. For each window we collected 1500 image pairs with time difference between frames of 210 μs at 1 Hz frequency. Every pair is processed with a multi-pass PIV algorithm (LaVision). We used an initial interrogation window of 96 × 96 pixel^2^ and a final pass with 48 × 48 pixel^2^ with a 50% of overlap, which results in a vector separation of 0.4 mm.

## Results

**Fig. 3:**
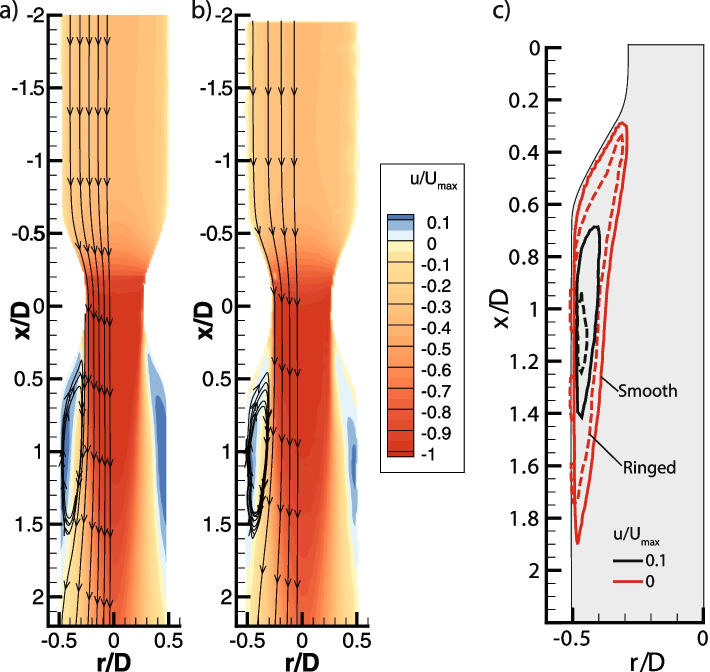
Velocity contours for streamwise direction for **a** smooth model and **b** model with cartilaginous rings (normalized by the maximum velocity in the contraction). **c** Contour line comparison of both models. It can be observed that the area with reversed flow is significantly larger in the smooth case, particularly that with u/Umax > 0.1

Velocity contours were obtained from the 2D-PIV experiments for both the smooth and the ‘ringed’ models, as shown in Fig. 3. In our analysis, *x* and *r*, represent the streamwise and radial directions with corresponding velocities *u* and *v* and velocity fluctuations *u’* and *v’*. Velocity fluctuations were obtained by subtracting the instantaneous velocity at each instant from the temporal mean velocity at each location in the domain. Since the models are axisymmetric, the same behavior is expected along the azimuthal direction. For both models, an acceleration of the flow is noted at the contraction, as expected from the decrease of cross-sectional area, and at the expansion flow separation occurs, as was also reported by Brouns et al. [7]. The adverse pressure-gradient at the expansion of the cross-sectional area after the stenosis, triggers the flow separation. Similar velocity is observed before the contraction in both models and a similar acceleration occurs at the contraction; however, the most notable difference between cases is the flow separation observed after the contraction (lower region). Notice, that the smooth model has a bigger and stronger flow separation than the model with rings. As can be observed in Fig. 3, the smooth separation region is darker than the ‘ringed’ one.

**Fig. 4:**
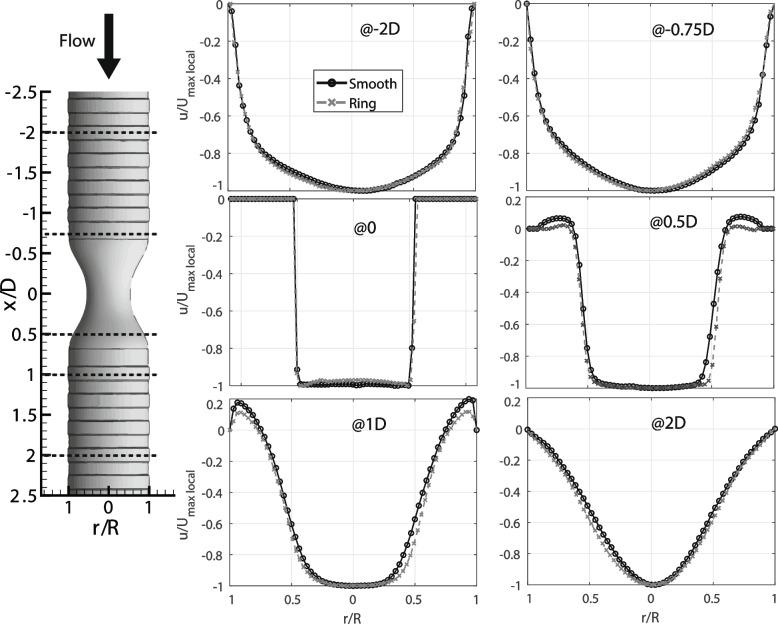
Velocity profiles from PIV results at five different locations along the trachea model (normalized by the maximum local velocities of the profile). The error bars, calculated as the standard error = (urms/Umax, local)/√N, where N is the number of samples, are of size similar to the symbols and have been omitted for clarity

To better understand the effect of the cartilaginous rings, we analyzed the velocity profiles of both cases (Fig. 4). These profiles are extracted at five different locations. From the upstream side of the contraction we have an inlet position two diameters (−2*D*) before the throat of the contraction and another at − 0.75*D.* In the downstream side of the contraction, the velocity is plotted at 0.5*D*, 1*D* and 2*D* to study the differences in the flow separation. From both profiles before the contraction, we can observe that the flow is practically the same for both cases, which rules out differences due to inlet conditions. Nevertheless, the flow after the contraction is different in both cases. The velocity profiles dependence on the position with respect to the rings has been presented elsewhere [27].

As noted from the velocity contours in Fig. 3, the separation region is stronger for the smooth case. In the model with cartilaginous, a smaller separation bubble at 0.5*D* and at 1*D* can be observed. At 0.5*D,* the separation bubble of the model with rings is 11.5% smaller than the smooth model, which implies that the separation is occurring earlier in the smooth model. The size of the recirculation bubble is quantified by the area with positive (against streamwise) velocity. Our results indicate a reduction in the separation bubble area of 26% with respect to the smooth case. After the contraction, at 1*D* the separation continues to be smaller for the ‘ringed’ model. Along with the differences in size, the intensity of the recirculation is also distinct. The effect of the rings reduces the maximum upstream velocity at the separation region by 38%. Similar results of recirculation reduction have also been reported in the separation region at the bifurcation from the trachea to the bronchi [6, 27].

**Fig. 5:**
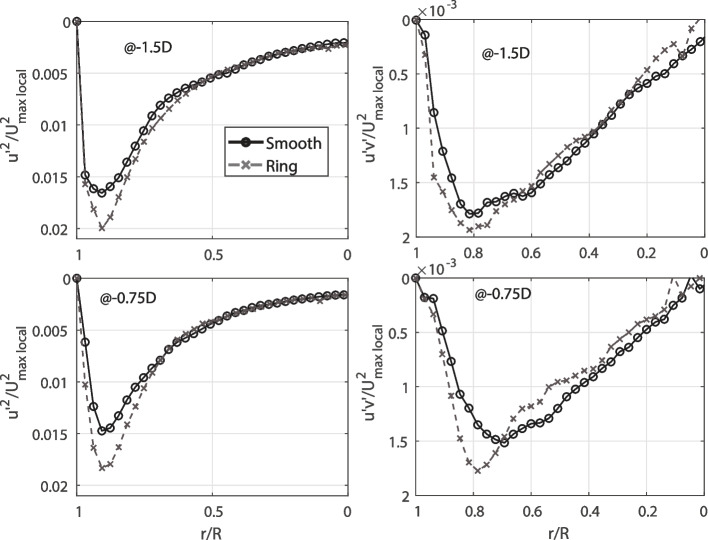
Reynold stresses upstream of the contraction (normalized by the maximum local velocity). The standard error of the Reynolds stresses is calculated as S.E. (u’2/U2) = (u’2/U2)/√(2 N - 2); with *N* = 1500. This gives a value of about or 1.8%, which is two orders of magnitude smaller than the values at the peak, making even small differences statistically significant

In order to understand the phenomena producing these differences in the flow separation between models, we observe with more detail the upstream side of the contraction. Although the mean velocity profiles between models are very similar (Fig. 4), the separation regions are very distinct. Hence, we observe the velocity fluctuation, that is, the turbulent component of the velocity field, occurring near the walls of the models. Small bubbles trapped before the contraction caused light reflections on the top of the model (in the experiment the model is hold horizontally). Hence, we are not able to obtain an accurate value of fluctuations on the top. However, the lower side of the model did not have reflections and we obtained the values of fluctuations at 1.5 diameters (− 1.5*D*) before the contraction and at − 0.75*D*. These results are presented in Fig. 5. We evaluate the streamwise Reynolds stresses (*u’*^*2*^*/U*^*2*^_*max local*_) and the Reynolds shear stresses (*u’v’/U*^*2*^_*max local*_), both normalized by the maximum local velocity at the centerline.

The production term (*P*) of turbulent kinetic energy (TKE = *u*’^2^/2 + *v*’^2^/2) is defined by the Reynolds shear stresses and the gradient of the mean streamwise velocity (*∂U/∂y*):

$$P={u}^{\prime }{v}^{\prime}\frac{\partial U}{\partial y}.$$

**Fig. 6:**
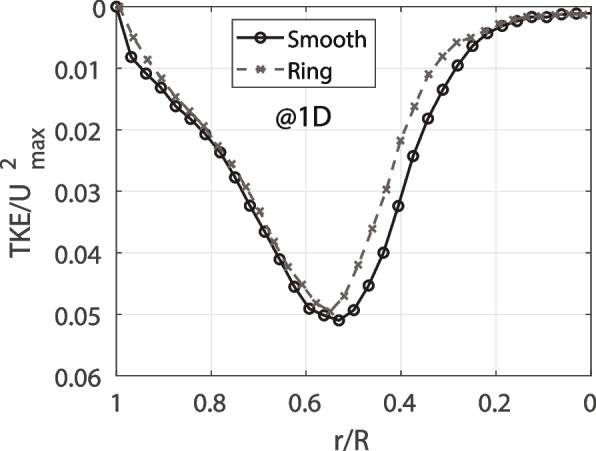
TKE at one diameter (1D) after the contraction (normalized by the maximum velocity at the contraction of the model)

As observed in Fig. 5, both the normal Reynolds stresses and turbulence production differences between the models increase as the flow develops along the downstream direction. At − 1.5*D,* the peak of the streamwise fluctuation is increased by 19% because of the rings, which occurs after going over three rings. Closer to the contraction at − 0.75*D* the difference at the peak is increased to 23%. Likewise, the production of turbulence increased by 7% at − 1.5*D* and 16% at − 0.75*D*. The smooth model fluctuations stay constant along the model, contrary to the ‘ringed’ model, where the turbulence increases. This occur because the rings are generating perturbations to the flow near the wall, as was also reported in the tracheobronchial flow study [6]. Finally, as observed in Fig. 6, the TKE is reduced in the case of the ‘ringed’ model, where the TKE downstream of the contraction is reduced by 3%.

## Discussion

In previous studies, it has been demonstrated that cartilaginous rings reduce the separation at the bifurcation regions of the trachea [6, 27]. It was speculated that the rings are inducing disturbances near the wall region which increase the momentum flux toward the wall in the separation region. In this study, we have detected a decrease of the flow separation consistent with previous studies on tracheobronchial flow. However, here we present evidence of the cartilaginous rings disturbing the flow and creating fluctuations and turbulence production near the wall. Such perturbations caused by the cartilaginous rings, transition the boundary layer to a turbulent regime, increasing the momentum flux toward the wall. Consequently, the increase of momentum flux over the ringed surface reduces separation compared to the smooth wall case [8]. As the cartilaginous rings reduce the separation, it also reduces the strength of the shear layer generated along the separation bubble’s edge, therefore reducing the Reynolds stresses and turbulence downstream of the contraction.

As the flow develops along the model with rings, the fluctuations increase and reduce the separation in the flow. Since the size and strength of the separation bubble is reduced, the shear layer is also reduced, which reduces leads to less mixing in the flow and, consequently, less turbulence.

In an analysis of pipe flow over periodic surface roughness it was determined that the presence of such surface modifications leads to periodic fluctuations on the flow [37]. Similarly, in our model we observe an increase of fluctuations. Although we did not perform pressure measurements, we can expect a bigger pressure drop along the trachea with cartilaginous rings due to increased turbulent losses. However, the length of the trachea is small and would not have a considerable effect. Nevertheless, the fluctuations generated by the rings are strong enough to cause a considerable difference on the flow separation, leading to possible escalated effects on the consequent branching generations in the respiratory tract.

As the flow near the wall becomes more turbulent, it promotes particle deposition locally as the fluctuations increase [19]. Zhang and Finlay [44] reported an increase in particle deposition along the trachea but saw little effect in the deposition at the bronchi. Similarly, Russo et al. [33] found particle deposition increase along the trachea but not considerable differences at the bifurcation regions. By reducing the separation region, the particles will be less prone to be deposited at the separation regions; though, the higher turbulence downstream will consequently increase the particle deposition downstream.

As previously mentioned, Brouns et al. [7] found that pressure drop was only found under severe constriction, which makes the diagnosis for cases with constriction greater than 70% difficult. The delayed separation induced by the rings may reduce the pressure drop even further, which could be a likely explanation for the difficulty in the diagnosis of trachea stenosis. In addition, the particle deposition in trachea stenosis cases can be affected by the decrease of flow separation, since it will have less separation area and less mixing downstream [20]. Such effect can also be related to the findings of Bocanegra Evans and Castillo [6], where less vorticity was found for the ringed case due to the reduction in flow separation at the bifurcation.

It is well-established that the geometrical characteristics of the roughness (height, pitch, width) modify its interaction with the flow [16]. Here, we have focused on the typical geometry of the cartilaginous rings, trachea and constriction to determine whether the presence of rings affects the behavior of the flow, but it is expected that the results will depend on the geometry and the flow characteristics.

## Conclusion

We analyzed the effect of the cartilaginous rings in a trachea model with stenosis (70% area contraction). A comparison between two models, one with a smooth wall and one with idealized cartilaginous rings, is carried out. We observed similar mean flow fields before the contraction, with the main velocity differences found downstream of the contraction: the separation region was reduced considerably in the ‘ringed’ model. The cartilaginous rings perturbed the flow near the wall by increasing the velocity fluctuations, hence delaying the separation at the expansion. The physical mechanism that reduces the flow separation is explained by the increase of momentum flux toward the wall resulting from an increased turbulent kinetic energy near the wall.

The most important observation from these results is that even though minor differences were observed in the mean velocity field, the rings produce perturbations that generate larger velocity fluctuations (and consequently Reynolds stresses). These results highlight the importance of cartilaginous rings and other small features along the airway wall when studying respiratory fluid dynamics. While our model is a simplified geometry, compared to the actual human respiratory system complex geometry, our results allow us to isolate the effect that cartilaginous rings have on the flow evolution. Future studies should focus on the effect of the delayed separation in subsequent generations to better understand how these flow perturbations propagate along the respiratory system.

## Data Availability

The datasets during and/or analysed during the current study available from the corresponding author on reasonable request.

## Abbreviations

APG: Adverse Pressure Gradient
CT: Computer Tomography
PIV: Particle Image Velocimetry
PDMS: Polydimethylsiloxane
PVA: Polyvinyl Alcohol
TKE: Turbulent Kinetic Energy
